# The GDNF System Is Altered in Diverticular Disease – Implications for Pathogenesis

**DOI:** 10.1371/journal.pone.0066290

**Published:** 2013-06-21

**Authors:** Martina Böttner, Martina Barrenschee, Ines Hellwig, Jonas Harde, Jan-Hendrik Egberts, Thomas Becker, Dimitri Zorenkov, Karl-Herbert Schäfer, Thilo Wedel

**Affiliations:** 1 Institute of Anatomy, Christian-Albrechts-University of Kiel, Kiel, Germany; 2 Department of General and Thoracic Surgery, University Hospital Schleswig-Holstein Campus Kiel, Kiel, Germany; 3 Department of Neurology, University Hospital Schleswig-Holstein Campus Kiel, Kiel, Germany; 4 Department of Informatics and Microsystems Technics, University of Applied Sciences, Kaiserslautern, Germany; Alexander Flemming Biomedical Sciences Research Center, Greece

## Abstract

**Background & Aims:**

Absence of glial cell line-derived neurotrophic factor (GDNF) leads to intestinal aganglionosis. We recently demonstrated that patients with diverticular disease (DD) exhibit hypoganglionosis suggesting neurotrophic factor deprivation. Thus, we screened mRNA expression pattern of the GDNF system in DD and examined the effects of GDNF on cultured enteric neurons.

**Methods:**

Colonic specimens obtained from patients with DD (n = 21) and controls (n = 20) were assessed for mRNA expression levels of the GDNF system (GDNF, GDNF receptors GFRα1 and RET). To identify the tissue source of GDNF and its receptors, laser-microdissected (LMD) samples of human myenteric ganglia and intestinal muscle layers were analyzed separately by qPCR. Furthermore, the effects of GDNF treatment on cultured enteric neurons (receptor expression, neuronal differentiation and plasticity) were monitored.

**Results:**

mRNA expression of GDNF and its receptors was significantly down-regulated in the muscularis propria of patients with DD. LMD samples revealed high expression of GDNF in circular and longitudinal muscle layers, whereas GDNF receptors were also expressed in myenteric ganglia. GDNF treatment of cultured enteric neurons increased mRNA expression of its receptors and promoted neuronal differentiation and plasticity revealed by synaptophysin mRNA and protein expression.

**Conclusions:**

Our results suggest that the GDNF system is compromised in DD. In vitro studies demonstrate that GDNF enhances expression of its receptors and promotes enteric neuronal differentiation and plasticity. Since patients with DD exhibit hypoganglionosis, we propose that the observed enteric neuronal loss in DD may be due to lacking neurotrophic support mediated by the GDNF system.

## Introduction

Diverticular disease (DD) is a widespread disease in industrialized countries with increasing prevalence in the elderly. The initial pathology is characterized by multiple mucosal/submucosal outpouchings throughout the colonic muscle coat which may lead to a broad spectrum of symptoms with potentially lethal complications [1]. Despite the considerably high prevalence, basic research addressing the pathogenesis of DD is lacking behind and the underlying cellular and molecular mechanisms remain largely unknown [2].

Traditionally, low fiber diet, elevated body mass index and connective tissue alterations are considered as risk factors for diverticulitis [3], [4]. More recently, novel pathophysiological concepts have addressed alterations of the enteric nervous system (ENS) associated with diverticulitis and the generation of symptoms in chronic DD [5], [6] Previous reports and data from our group have given evidence for an underlying enteric neuropathy in diverticulitis characterized by a decrease of myenteric nerve cells (oligoneuronal hypoganglionosis) and reduced nerve fibers within smooth muscle layers [7], [8], [9], [10] It is postulated that the disturbed intestinal innervation pattern gives rise to colonic motility disorders frequently reported in DD [11] thereby promoting the development of diverticula. Although a loss of enteric neurons represents a common histopathologic phenotype within the spectrum of gastrointestinal neuromuscular pathology (GINMP) [12], the reason for the reduced ganglionic nerve cell content observed in DD remains unclear.

Survival, differentiation and maintenance of enteric neurons are strongly influenced by neurotrophic factors. Glial cell line-derived neurotrophic factor (GDNF) is a key neurotrophin for the ENS isolated originally from the supernatant of the glial cell line B49 and characterized by its ability to promote the survival of cultured dopaminergic neurons [13]. GDNF is a member of the TGF-ß superfamily of growth factors which regulate numerous functions in the development and differentiation of the nervous system [14]. The GDNF-induced signal transduction is mediated via the glycosyl phosphaditylinositol-anchored receptor GDNF family receptor α1 (GFRα1) and the rearranged during transfection (RET) receptor tyrosine kinase [15]. The impact of the GDNF system on the ENS became evident, when gene-ablated animal models were analyzed for ENS defects. Deletion of GDNF leads to total intestinal aganglionosis, i.e. the complete loss of enteric neurons in the small and large intestine [16]. A similar gross phenotype is observed in mice ablated for the GDNF receptors GFRα1 or RET underlining the essential role of the GDNF system in the development and maintenance of the ENS [17], [18].

As GDNF-ablated mice exhibit total intestinal aganglionosis and DD is associated with intestinal hypoganglionosis, we raised the question whether the GDNF system might be compromised in DD thereby contributing to the partial loss of enteric neurons. Therefore, the tissue sources expressing GDNF and its receptors were identified in the human colon and gene expression profiles of the GDNF system were compared between patients with DD and controls. Moreover, the effects of GDNF on GDNF receptor expression as well as on the differentiation and plasticity of enteric neurons were monitored in rat postnatal enteric nerve cell cultures.

## Materials and Methods

### Patients

#### Control group

Segments of sigmoid colon were obtained from patients (n = 20, 9 females, 11 males, mean age: 71.3 years) who underwent anterior rectal resection for non-obstructive colorectal carcinoma. Anorectal evacuation and colonic motility disorders as well as the presence of colonic diverticula were previously excluded. Full-thickness specimens were harvested at safe distance (>5 cm) from the tumor and immediately transferred from the operating room to the laboratory for tissue processing. The study of human tissue received approval from the Local Ethics Committee of the Faculty of Medicine, Christian-Albrechts-University of Kiel, Germany (B299/07).

#### Patients with DD

Segments of sigmoid colon were obtained from patients (n = 21, 14 females, 7 males, mean age: 60.8 years) who underwent sigmoid resection or left hemicolectomy for symptomatic DD. Patients were operated after two or more attacks of diverticulitis by elective surgery. Full-thickness specimens were harvested from sites adjacent to colonic diverticula. Diverticula-containing areas displaying an altered anatomy of the colonic wall due to transmural mucosal/submucosal outpouchings or signs of inflammation and fibrotic scaring were excluded from tissue sampling. The specimens were immediately transferred from the operating room to the laboratory for tissue processing. The study of human tissue received approval from the Local Ethics Committee of the Faculty of Medicine, Christian-Albrechts-University of Kiel, Germany (B299/07).

### Tissue Preparation

#### Tissue processing for mRNA expression profiles of the muscularis propria

The muscularis propria was isolated from full-thickness biopsies of the colonic wall, immediately frozen in isopentane and stored at −70°C until use. Prior to RNA isolation 20 orthogonal cryosections (10 µm) were cut on a cryostat and collected in RNA lysis buffer (Qiagen, Hilden, Germany).

#### Tissue processing for mRNA expression profiles of LMD samples

Full-thickness biopsies (2 cm border length) were immediately frozen in isopentane and stored at −70°C until use. Orthogonal cryosections (10 µm) were placed on membrane-coated (polyethylene naphtalate, 1.0 µm, Zeiss, Göttingen, Germany) slides. To visualize myenteric ganglia sections were ultra-rapidly (ca. 60 s) stained with toluidine blue and air-dried.

### Laser Microdissection and Pressure Catapulting

Laser-microdissection (LMD) was performed by a modified method described previously [19]. Briefly, myenteric ganglia and smooth muscle cells of the muscularis propria were identified by inverse lightmicroscopy (Zeiss Axio Observer Z1, Zeiss, Göttingen, Germany), excised by laser microdissection and collected by laser pressure catapulting (PALM MicroLaser System, Zeiss, Göttingen, Germany) in caps of 0.5 ml reaction tubes. Ganglionic and smooth muscle tissue areas of 2 mm^2^ were collected, immediately dissolved in 350 µl RNA lysis buffer (Qiagen, Hilden, Germany) and stored at −70°C until further use.

### Enteric Nerve Cell Culture

Preparation of myenteric ganglionic cells was performed according to a method described previously [20], [21]. Briefly, the small intestine was removed from newborn Wistar rats (postnatal day 2–3), the muscularis propria was stripped from the mucosa und placed in Ca2+- and Mg2+-free Hankś Balanced Salt Solution (HBSS, Gibco Life Technologies, Germany) with antibiotics containing 1 mg/ml collagenase (SIGMA, Munich, Germany). After 2 h of incubation at 37°C fragments of the myenteric plexus were collected under stereomicroscopic control and dissociated by digestion with trypsin/EDTA (0.125 mg/ml Gibco, Life Technologies, Germany) for 15 min at 37°C. The cells were harvested and centrifuged at 900 rpm. Trypsination was stopped by replacing it with fetal calf serum (FCS, Gibco, Life Technologies, Germany). The cells were triturated, counted and seeded in a density of 100.000 cells/ml on poly-D-Lysin-(SIGMA)/Laminin- (SIGMA, Munich, Germany) coated coverslips for immunocytochemistry studies or 12-well-plates for gene expression studies. The defined medium for incubating the cells consisted of Neurobasal A (Gibco, Life Technologies, Germany) and B27 supplement (Gibco, Life Technologies, Germany). GDNF (Peprotech, Hamburg, Germany) was added to a final concentration of 2, 10, or 50 ng/ml. For gene expression analysis (GDNF receptors, synaptophysin) and morphometric studies (neuronal counts and network formation) cells were cultured for 1 week, for immunocytochemical detection of synaptophysin culture time was 3 weeks. Medium was changed every second day.

### RNA Extraction and Reverse Transcription

Extraction of total RNA from human tissue was performed using a Nucleospin II kit (Macherey and Nagel, Düren, Germany), RNA from enteric nerve cell cultures was isolated using a Nucleospin XS kit (Macherey and Nagel, Düren, Germany) according to the manufactureŕs guidelines. Prior to reverse transcription, contaminating genomic DNA was digested in a volume of 15 µl using 1.5 U of DNAse I (Sigma, Munich, Germany). Reverse transcription was carried out in a total volume of 30 µl containing 200 ng RNA, 375 ng random hexamer primer (GE Healthcare, Freiburg, Germany), 0.5 mM dNTPs (Promega, Mannheim, Germany), 0.01 M DTT, 1×reaction buffer, and 150 U Superscript II Reverse Transcriptase (Invitrogen, Karlsruhe, Germany). The annealing, elongation, and denaturation steps were carried out at 25°C for 10 min, at 42°C for 50 min, and at 70°C for 15 min, respectively.

### Quantitative PCR

Quantitative PCR (qPCR) reactions were run on an ABI Prism 7700 Sequence Detection System (TaqMan, Applied Biosystems, CA, U.S.A.). Amplification reactions were carried out in a 20 µl volume containing 1×qPCR Master Mix Plus (Eurogentec, Cologne, Germany), 900 nM primers, 225 nM hybridization probe, and 2 µl cDNA. Samples were run in duplicate and amplified over 50 cycles. Each cycle consisted of a denaturation phase of 15 s at 95°C and a hybridization/elongation phase of 1 min at 60°C. mRNA expression profiles were measured for GDNF, GFRα1, RET, synaptophysin, and the housekeeping gene HPRT in human samples, and for GFRα1, RET, synaptophysin and HPRT in rat enteric cell cultures. Forward and reverse primers and probes are listed as supporting document Table S1.

### Immunocytochemistry of Enteric Nerve Cell Cultures

#### Immunocytochemistry for morphometric analysis

Cells were fixed for 30 min with 4% paraformaldehyde, permeabilized for 10 min with methanol and treated for 10 min with 3% H_2_O_2_. Following blocking of unspecific signals with normal goat serum (1∶10) for 30 min, primary antibodies diluted in antibody diluent (Zymed, Invitrogen, CA) were applied for 1 h: mouse anti-HuC/D (1∶500, Molecular Probes, Invitrogen) or mouse anti-tubulin ßIII, (1∶200, Merck Millipore, Darmstadt, Germany). HuC/D is a pan-neuronal marker staining specifically neuronal somata thereby allowing reliable identification and counting of nerve cells. Tubulin ßIII is a microtubular protein allowing reliable identification of neuronal processes and evaluation of the nervous network. After incubation with the secondary antibody, biotinylated goat anti-mouse IgG (1∶200, Jackson Immuno Research, PA) for 30 min and ABC conjugated with horseradish peroxidase were applied for 30 min. DAB (DakoCytomation, Hamburg, Germany) was used as chromogen. Analysis was carried out with a Zeiss Axiophot microscope (Zeiss, Göttingen, Germany).

#### Dual-label immunocytochemistry

After fixation and permeabilization as described above, samples were incubated with a rabbit anti-synaptophysin antibody (1∶1000, Biozol, Echingen, Germany) and a mouse anti-PGP 9.5 antibody (1∶1000, Acris, Herford, Germany) for 1 h. After 30 min incubation with the secondary antibodies, goat anti-rabbit-AlexaFlour488 (1∶250, Invitrogen, Karlsruhe, Germany) and goat-anti-mouse-AlexaFluor546 (1∶250, Invitrogen, Karlsruhe, Germany), cells were counterstained with DAPI (Roche, Mannheim, Germany) to visualize cell nuclei. Analysis was carried out with a fluorescent microscope (Axiovert 200M, Zeiss, Göttingen, Germany) coupled to a digital camera (Axiocam, Zeiss, Göttingen, Germany). Data acquisition was performed with Axiovision software (Zeiss, Göttingen, Germany).

### Morphometric Analysis of Enteric Nerve Cell Cultures

#### Technical devices, software

Morphometric analysis was carried out by using a light microscope (Axiophot, Zeiss, Germany) coupled to a digital camera (Axiocam, Zeiss, Germany). The software program Axiovision (version 4.7, Zeiss, Germany) was used for analysis. The data were transferred into Excel software (version 9.0) and further processed for statistical analysis and data plotting (PrizmTM, GraphPad, Sand Diego, CA, USA).

#### Nerve cell counts

The number of HuC/D-immunoreactive nerve cells was analyzed per optical field (objective with 20× magnification). Neurons were recorded and counted in three randomly assigned optical fields and the mean value was calculated. Experiments were carried out in quadruplicate (50 ng/ml GDNF vs. control).

#### Neuronal network area

The area occupied by the tubulin ßIII-immunoreactive neuronal network was measured and related to the overall-area of the optical field (objective with 20× magnification). Five randomly chosen optical fields were analyzed using an area-analysis-tool (AnalySIS Pro 3.1, Soft Imaging Software, Münster, Germany) to allow reliable depiction of the entire nervous network. The mean value was calculated and expressed as relative area related to the optical field (percentage). Experiments were carried out in triplicate (50 ng/ml GDNF vs. control).

### Statistical Analysis

Comparison of mRNA expression levels of the GDNF system between the control group and patients with DD as well as morphometric analysis of enteric nerve cell cultures comparing GDNF-treated cultures vs. controls were carried out by using non-parametric Mann-Whitney U-tests. The mRNA expression of the GDNF system in laser-microdissected myenteric ganglia and muscle layers and the effects of the GDNF treatment on rat enteric nerve cell cultures regarding gene expression studies were analyzed by one-way-ANOVA followed by Newman-Keuls post hoc test (PrizmTM, GraphPad, Sand Diego, CA, USA). Differences were considered significant if p<0.05.

## Results

### Gene Expression Profiles of the GDNF System in Patients with DD and Controls

**Figure 1:**
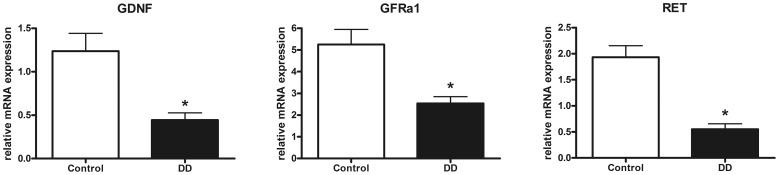
mRNA expression of the GDNF system in the muscularis propria of the human colon. mRNA expression of GDNF (A) and its receptors GFRα1 (B) and RET (C) is significantly down-regulated in patients with DD compared to controls. mRNA levels are determined by qPCR, expression of target genes is normalized to mRNA expression of the house-keeping gene HPRT. Data are shown as mean +/− SEM, n = 20 (controls) and n = 21 (DD), *p<0.05 vs. control.

To determine the regulation of the GDNF system in patients with DD compared to controls mRNA expression levels of GDNF and its corresponding receptors GFRα1 und RET were monitored by qPCR. Analysis of the muscularis propria revealed that the GDNF mRNA expression was strikingly down-regulated in patients with DD (p<0.001), as mRNA levels dropped to 36% of control values (Fig. 1A). The GDNF receptor GFRα1 was down-regulated to 48% of control values (p<0.001, Fig. 1B) and RET mRNA levels dropped to 29% of mRNA expression detected in controls (p<0.001, Fig. 1C).

### Tissue Source of Components of the GDNF System in the Human Colon

**Figure 2:**
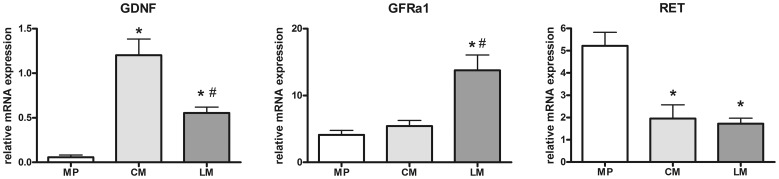
Site-specific mRNA expression of the GDNF system in the human colon. mRNA levels of GDNF (A), GFRa1 (B), and RET (C) in myenteric plexus (MP) and in the circular (CM) and longitudinal (LM) smooth muscle layer isolated by LMD. The main source for GDNF mRNA are the smooth muscle layers, whereas the GFRα1 is expressed mainly in the longitudinal muscle layer but also in myenteric plexus and RET is predominantly expressed by myenteric plexus and to a lesser extent by smooth muscle layers. mRNA expression levels are normalized to mRNA expression of the house-keeping gene HPRT. Data are shown as mean +/− SEM. GDNF: n = 10 (MP), n = 15 (CM), n = 14 (LM), GFRa1: n = 10 (MP), n = 15 (CM), n = 15 (LM), RET: n = 10 (MP), n = 15 (CM), n = 14 (LM). *p<0.05 vs. MP, #p<0.05 vs. CM.

To identify the tissue source of components of the GDNF system the intestinal compartments of interest were selectively isolated by LMD. Site-specific gene expression profiles of GDNF and its receptors GFRα1 and RET were monitored in myenteric ganglia as well as in the circular and longitudinal smooth muscle layers. GDNF mRNA levels were signifcantly higher in the circular (p<0.001) and longitudinal (p<0.05) muscle compared to the myenteric plexus. (Fig. 2A). Highest GDNF expression was found within the circular muscle. GFRα1 was present in all intestinal compartments investigated. Whereas levels in the myenteric plexus and the circular muscle were similar, mRNA expression in the longitudinal muscle exceeded significantly that observed in the myenteric plexus (p<0.001) and circular muscle (p<0.001) (Fig. 2B). RET mRNA expression was significantly higher in the myenteric plexus than in the circular (p<0.001) and longitudinal muscle (p<0.001) (Fig. 2C).

### Effects of GDNF on GDNF Receptor Expression in Enteric Nerve Cell Cultures

**Figure 3:**
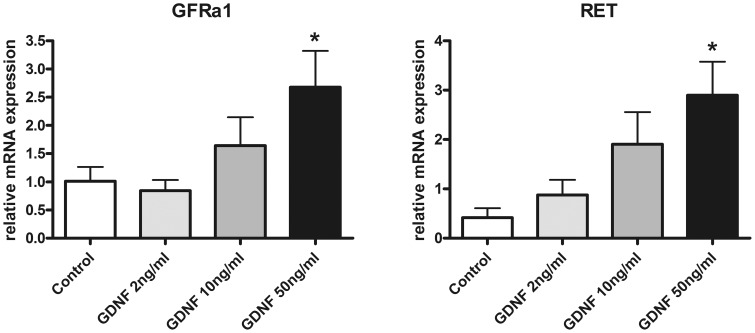
mRNA expression of GDNF receptors in enteric nerve cell cultures following GDNF treatment. Treatment with GDNF for 1 week increases mRNA expression of GDNF receptors GFRα1 (A) and RET (B) in enteric nerve cell cultures. mRNA expression levels are normalized to mRNA expression of the house-keeping gene HPRT. Data are shown as mean +/− SEM, n = 14–15 per experimental group, *p<0.05 vs. control.

As we aimed to investigate the putative effect of a reduced GDNF mRNA expression on enteric neurons, we implemented a cell culture model of rat postnatal dissociated myenteric plexus exposed to increasing concentrations of GDNF. First, the effect of GDNF on mRNA expression of its corresponding receptors GFRα1 and RET was assessed. After 1 week of GDNF treatment, both receptors showed up-regulation of their respective mRNA expression (Fig. 3). A dose of 50 ng/ml GDNF increased GFRα1 mRNA levels 2.6 fold compared to controls (Fig. 3A), and RET mRNA expression was up-regulated 7.0 fold compared to untreated cell cultures (Fig. 3B). These findings indicate that the growth factor GDNF is able to induce mRNA expression of its receptors responsible for mediating neurotrophic effects.

### Effects of GDNF on Neuronal Differentiation in Enteric Nerve Cell Cultures

**Figure 4:**
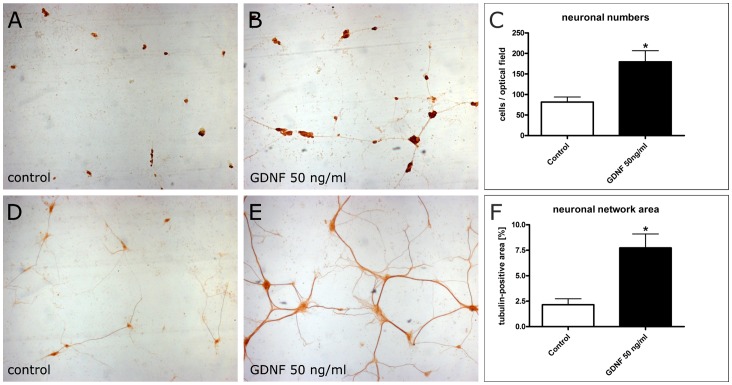
Effects of GDNF on neuronal number and differentiation of enteric nerve cell cultures. Rat enteric nerve cells were cultured for 1 week without (A, D) or with 50 ng/ml GDNF (B, E). Immunocytochemistry for the neuronal marker HuC/D (A, B) shows that neuronal aggregates are more numerous and larger after GDNF treatment. The morphometric analysis (C) confirms the increased neuronal number following GDNF treatment. Immunocytochemistry for tubulin ßIII (D, E) visualizes neuronal processes which are more densly distributed and ramified after GDNF treatment. The morphometric analysis (F) confirms the increased area of the neuronal network following GDNF treatment. Magnification 4×. Data are shown as mean +/− SEM, n (HuC/D immunocytochemistry) = 11–12, n (ßIII tubulin immunocytochemistry) = 7–8 per experimental group, *p<0.05 vs. control.

The neurotrophic capacity of GDNF was studied by assessing the neuronal number and differentation of the neuronal network in enteric nerve cell cultures exposed to GDNF. The rationale of these experiments was to conclude whether a lack of GDNF may contribute to the relative loss of enteric nerve cells observed in patients with DD. The numerical increase of cultured myenteric neurons was monitored by anti-HuC/D immunocytochemistry. After a culture time of 1 week, few dispersed HuC/D immunoreactive nerve cells were observed in control cultures (Fig. 4A). At the same time, neuronal numbers were significantly (p<0.01) elevated following treatment with 50 ng/ml GDNF (Fig. 4B). Morphometric analysis revealed that GDNF treatment led to a 2.2 fold increase of nerve cells compared to controls (Fig. 4C). Differentiation of the neuronal network was monitored by anti-tubulin ßIII immunocytochemistry. After a culture time of 1 week, control cultures displayed only small-sized neuronal aggregates with few and thin interconnecting nerve fiber strands (Fig. 4D). In contrast, following GDNF treatment neuronal aggregates were larger and the interconnecting nervous network displayed densely ramified nerve fiber strands of markedly increased thickness (Fig. 4E). Morphometric analysis revealed that GDNF treatment led to a 3.6 fold increase (p<0.01) of the neuronal network area compared to controls (Fig. 4F).

### Effects of GDNF on Neuronal Plasticity in Enteric Nerve Cell Cultures

**Figure 5:**
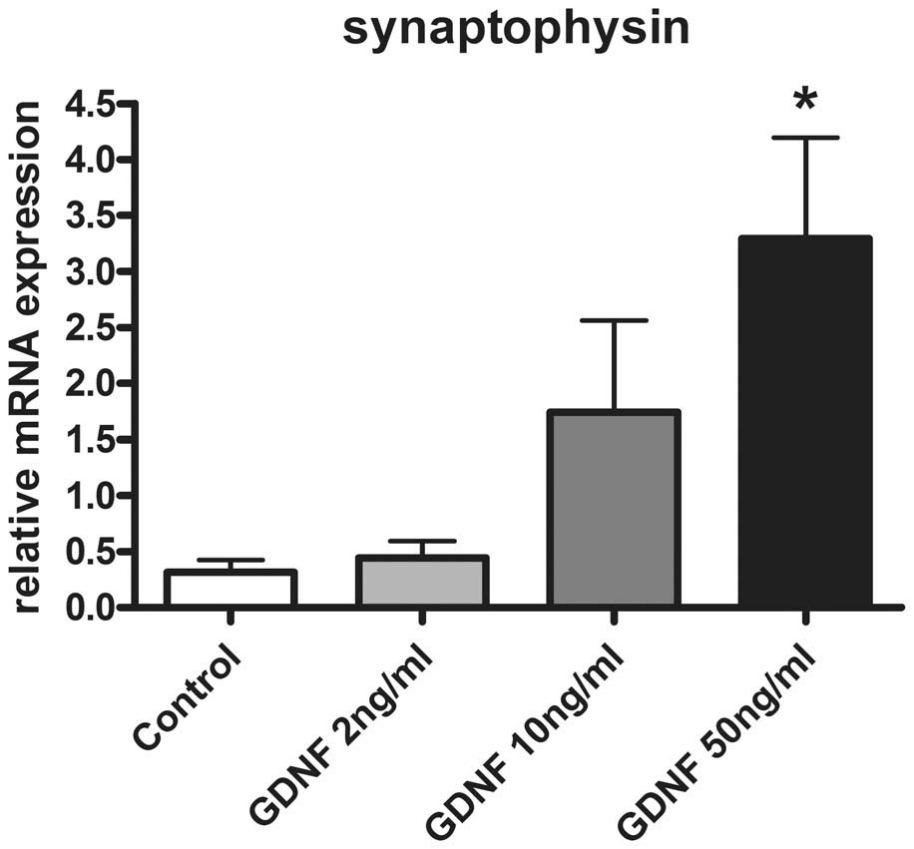
Synaptophysin mRNA expression of enteric nerve cell cultures after GDNF treatment. GDNF increases mRNA expression of synaptophysin in rat enteric nerve cell cultures. Expression levels were measured after one week and are normalized to expression of the house-keeping gene HPRT. Data are shown as mean +/− SEM, n = 11–12 per experimental group, *p<0.05 vs. control.

**Figure 6:**
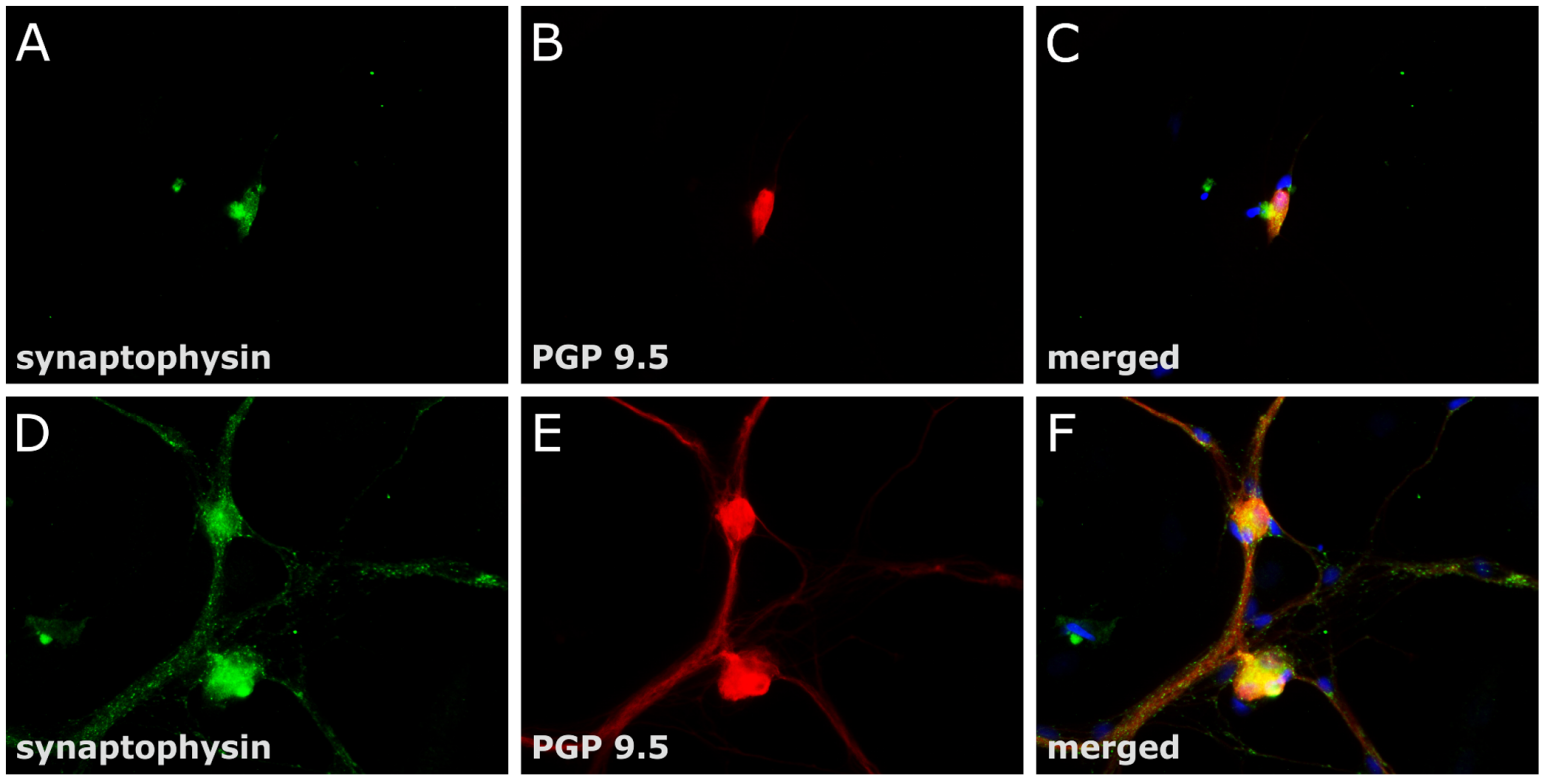
Synaptophysin immunoreactivity of enteric nerve cell cultures after GDNF treatment. Rat enteric nerve cells were cultured for 3 week without (A-C) or with 50 ng/ml GDNF (D-F). Dual label immunocytochemistry for synaptophysin (green, A, D) and the pan-neuronal marker PGP 9.5 (B, E) was performed. In the merged pictures (C, F) cellular nuclei are stained with DAPI (blue). Cell cultures treated with GDNF display punctuate and granular synaptophysin immunoreactivity along ramifying nerve fibers most likely resembling accumulated synaptic vesicles, whereas in untreated cell cultures immunoreactive signals were confined to neuronal somata. Magnification: 40×.

The positive effect of GDNF on neuronal differentiation of enteric nerve cell cultures was further addressed by assessing the influence of this neurotrophic factor on neuronal plasticity. Therefore, the effects of GDNF on the synaptic vesicle marker synaptophysin were studied in enteric nerve cell cultures. mRNA expression of synaptophysin was dose-dependently increased following GDNF treatment after one week of culture time (Fig. 5). At a dose of 50 ng/ml, GDNF augmented mRNA levels of the synaptic vesicle marker 10.5 fold (p<0.01) compared to controls. To monitor the topographical expression pattern of synaptophysin, enteric nerve cell cultures were analyzed after 3 weeks of GDNF treatment by dual label immunocytochemistry for synaptophysin and the pan-neuronal marker PGP 9.5 (Fig. 6). Synaptophysin immunoreactivity was detectable both in control cultures (Fig. 6A) and in GDNF treated cultures (Fig. 6D). However, whereas under control conditions the protein was confined to neuronal somata (Fig. 6 B–C), GDNF treatment led to immunoreactive signals present in neuronal cell bodies as well as in neuronal processes (Fig. 6 E–F). The nerve fiber strands displayed a punctuate and granular synaptophysin immunoreactivity most likely resembling nerve fiber varicosities with accumulated synaptic vesicles. The findings indicate that GDNF positively influences synaptic vesicle formation thereby promoting neuronal plasticity.

## Discussion

To our knowledge, this is the first study addressing neurotrophic factor deficits in patients with DD and investigating in vitro the underlying cellular and molecular mechanisms. The study reveals four important findings: (1) mRNA expression of GDNF and its receptors GFRα1 and RET is down-regulated in the muscularis propria of patients with DD; (2) the main source of GDNF is the intestinal smooth muscle, while RET is mainly expressed in enteric ganglia in which GFRα1 is also found; (3) GDNF up-regulates its corresponding receptors in enteric nerve cell cultures; (4) GDNF treatment promotes neuronal differentiation and plasticity of enteric nerve cell cultures.

### Down-regulation of GDNF in Diverticular Disease

DD is a gastrointestinal disorder with a high prevalence but a still poorly understood pathogenesis. Traditional pathogenetical concepts have advocated over decades the impact of a low-fiber diet as mayor risk factor for DD. Whereas Painter and Burkitt [22] claimed already 40 years ago that a Western diet low in fibres is positively correlated with the risk of developing DD, a recently published study by Peery et al. [23] showed that high, not low, fiber intake is associated with an increased risk of diverticulosis. These novel findings might lead to a re-addressing of putative factors involved in the pathogenesis of DD. In fact, increasing evidence is given that DD is associated with an enteric neuropathy [5], [6]. In line with this concept, we and others recently demonstrated that colonic specimens from patients with DD are characterized by an oligoneuronal hypoganglionosis, i.e. a loss of neurons in the ENS [7], [9], [10].

Since GDNF- and GDNF receptor-knock-out mice exhibit total intestinal aganglionosis demonstrating the crucial role of this growth factor for the development and maintenance of the ENS [16], [17], [18], we addressed the question whether the GDNF system might be compromised in a condition associated with enteric neuronal loss such as in DD. Indeed, expression levels of both GDNF and GDNF receptors were significantly down-regulated in the muscularis propria of patients with DD. The functional impact of GDNF insufficiency is underlined by a report from Shen et al. [24] who showed that GDNF haploinsufficiency resulted in a loss of enteric neurons in mice heterozygous for a GDNF null mutation. In addition, animal models of diabetic rats showed a reduced intestinal GDNF expression accompanied by a loss of enteric neurons [25]. Therefore, reduced GDNF mRNA levels might contribute to the loss of enteric neurons observed in DD.

Although all specimens studied from patients with DD were obtained from histologically un-inflamed areas showing no evidence for abscess formation, transmural inflammatory infiltrates nor fibrotic scarring, it cannot be excluded that recurrent inflammation might have contributed to alterations of the GDNF system in DD, as it has been previously shown that GDNF levels are modified in inflammatory bowel diseases (e.g. ulcerative colitis, Crohńs disease, infectious colitis) [26]. Whereas these inflammatory states showed induced up-regulation of GDNF concentrations in inflamed areas, GDNF was down-regulated in non-inflamed tissue of patients with Crohńs disease. Thus, our finding of reduced GDNF mRNA expression in non-inflamed areas of the colon of patients with DD could also represent a consequence of previous inflammation having caused damage to the ENS and/or enteric musculature.

### Site-specific Gene Expression Profiles of the GDNF System in the Human Colon

To investigate the cellular sources of the components of the GDNF system in the human colon, we applied LMD to those tissue compartments of interest followed by qPCR. The intestinal muscle layers could be identified as the main source of GDNF in the adult human colon, whereas RET was mainly expressed by enteric ganglia. GFRα1 mRNA expression exhibited highest levels in the longitudinal muscle but was also expressed in enteric ganglia. Thus, the so called “neurotrophic factor concept” originally postulated for the CNS might also apply for the ENS: The neurotrophic factor concept in its basic form envisages that innervated tissues produce a signal (neurotrophic factor) specifically directed towards the innervating neurons which express receptors for the target-derived neurotrophic factor for the selective limitation of neuronal death occurring during development [27], [28]. Translated into the present context, this would mean that the innervated tissue, i.e. the intestinal smooth muscle, produces GDNF which is acting on its corresponding receptors (GFRα1 and RET) expressed by innervating nerves, i.e. myenteric neurons, to maintain their survival in adulthood – as recently confirmed by Rodrigues et al. for the rat intestine [29]. Consequently, a decreased amount of neurotrophic factor supply as evidenced by significantly reduced mRNA levels of GDNF would result in a loss of enteric neurons.

### Effects of GDNF on its Corresponding Receptor Expression

To elucidate the role of GDNF on maintenance of enteric neurons, we addressed the effect of GDNF treatment on cultured myenteric neurons derived from postnatal rats. GDNF treatment increased mRNA levels of its corresponding receptors, i.e. at a dose of 50 ng/ml GDNF up-regulated mRNA levels of GFRα1 and RET in myenteric neurons. This observation is in accordance to the site-specific gene analysis of the GDNF system in the human colon by means of LMD showing that enteric neurons represent the main expression site of GDNF receptors. Thus, down-regulation of GDNF in the muscularis propria of patients with DD might result in lower receptor expression in the ENS thereby compromising the effects of the GDNF system. Similar findings have been obtained in animal studies and cell culture models of rodent gut components. In a culture model of rat intestinal muscle cells Rodrigues et al. [29] identified GDNF expression in cultured smooth muscle cells. Other studies demonstrated that RET is a marker for neural crest-derived cells detected first in ENS precursor cells during their rostro-caudal migration through the developing gut and subsequently in the myenteric and submucosal plexus [30], [31]. Similarly, GFRα1 mRNA has been detected in enteric neurons of the developing intestine [32]. Conditional ablation of GFRα1 even in late gestational stages caused widespread neuronal death in the colon [33] emphasizing the importance of the GDNF system in maintaining neuronal survival.

### Effect of GDNF on Neuronal Survival and Differentiation of Postnatal Neurons

GDNF treatment of enteric nerve cell cultures enhanced both neuronal numbers and the formation of an extensive nervous network after one week of culture demonstrating that GDNF effectively promotes the survival and differentiation of postnatal myenteric neurons. Consistent with these results, Rodrigues et al. could demonstrate after 72 h of incubation enhanced neuronal survival, axonal outgrowth and nerve fiber fasciculation induced by GDNF using co-cultures of neonatal rat myenteric neurons, smooth muscle and glial cells [29]. Similar effects have been reported in cell culture models of embryonic enteric neurons, in which a dose of 100 ng/ml GDNF promoted survival and proliferation of RET-expressing E14.5 neural crest cells [34]. According to the present data, other reports have demonstrated increased survival of myenteric neurons derived from rats of postnatal day 1 or 7 following GDNF treatment [35]. Since some enteric neuronal precursors do not withdraw from the cell cycle until postnatal day 14 [36], these cells might represent a pool of pluripotent cells in the postnatal gut responsible for the GDNF-induced increase in neuronal cell numbers. Alternatively, the survival promoting effect of GDNF might be due to a reduction of cell death as demonstrated for dopaminergic neurons of the substantia nigra [37] or motoneurons [38]. In addition, the observed GDNF induced formation of an extensive neuronal network in enteric nerve cell cultures is consistent with the induction of axonal growth in the peripheral nervous system mediated by GDNF [39]. These findings might delineate a GDNF-induced mechanism on regulating neuronal proliferation and differentiation in postnatal myenteric neurons that extends beyond the role of GDNF during development as demonstrated by knock-out animals [16], [40], [41]. The neurotrophic effect of GDNF on postnatal enteric neurons on the one hand and the decrease of GDNF in the muscularis propria of patients with DD on the other hand may provide a mechanism to explain the observed hypoganglionosis observed in this condition.

### Effect of GDNF on Enteric Neuronal Plasticity

In addition to neuronal proliferation and differentiation GDNF also promoted the formation of synaptic vesicles in enteric nerve cell cultures as demonstrated by the upregulation of the synaptic vesicle marker synaptophysin. Outgrowing neuronal processes displayed multiple granular accumulations of synaptophysin resembling sites of synaptic communications also known as autonomic nerve fiber varicosities. These observations are consistent with a report of Zeng et al. [42] who described enhanced synaptic communication by modulating potassium currents and response to serotonin of cultured myenteric neurons following GDNF treatment. The increased expression of the synaptic vesicle marker synaptophysin induced by GDNF might have fundamental impact on understanding the pathogenesis of DD as well as of other enteric neuropathies: Recent research work in several neurodegenerative disorders of the CNS has emphasized the importance of synaptic dysfunction as the initial event preceeding subsequent neurodegeneration [43]. This pathogenetic concept has given rise to the term “synaptopathies” applying for Alzheimeŕs and Parkinsońs disease as well as prion diseases, schizophrenia and autism [44]. Thus, it is intriguing to speculate whether deficiencies of the synaptic vesicle apparatus in the ENS may be associated with enteric neuropathies and contribute to the pathogenesis of intestinal motility disorders including DD.

### Conclusion

Taken together, our results demonstrate for the first time that DD is associated with a decrease of the neurotrophic factor GDNF. The in vitro data illustrate that GDNF promotes differentiation and synaptic plasticity of postnatal enteric neurons arguing for a role in the maintenance of the ENS during postnatal life. The compromised GDNF system in patients with DD may help to explain the shortfall of enteric neurons in this condition and further strengthens the hypothesis that the pathogenesis of DD may be linked – amongst other etiological factors – to an underlying enteric neuropathy.

## Supporting Information

Table S1 **Primer sequences.**
(DOCX)
