# Supplementary material for: The GDNF System Is Altered in Diverticular Disease – Implications for Pathogenesis
Source: PLoS One. 2013 Jun 21;8(6):e66290. doi: 10.1371/journal.pone.0066290 (PMC3689736; doi:10.1371/journal.pone.0066290)
Supplement: Table S1 — Primer sequences. (DOCX) [file pone.0066290.s001.docx]

*Primers amplifying human sequences*

Human GDNF: Forward primer: 5´-tgaaaccaaggaggaactgatttt-3´, reverse primer: 5´-gtcactcaccagccttctatttctg-3´, probe: 5´- tactgcagcggctcttgcgatgcag-3´

Human GFRα1: forward primer: 5´- tcgggcaatacacacctctgt-3´, reverse primer: 5´- cttggaggagcagccattga-3´, probe: 5´- tgaaaaagaaggtctcggtgcttcc-3´

human RET: forward primer: 5´- aaggagatggcaaagggatcac-3´, reverse primer: 5´- ttgatgtcttgggtctccacaa-3´, probe: 5´- aggaacttctccacctgctctccc-3´

human synaptophysin: forward primer: 5´- ggccacagacccagagaacat-3´, reverse primer: 5´- gtgttgagtcccgaggtcaca-3´, probe: 5´- caaggagatgcctgtctgccgcca-3´

human HPRT: forward primer: 5´-tgaacgtcttgctcgagatgtg-3´, reverse primer: 5´-ccagcaggtcagcaaagaattt- 3´, probe: 5´-tgggaggccatcacattgtagcc-3´

*Primers amplifying rat sequences*

rat GFRα1: forward primer: 5´- gcgaatttgcaggctcagaag-3´, reverse primer: 5´- gaggcaccagcgagaccat-3´, probe: 5´-tgaaatccaatgtgtcgggtagca-3´

rat RET: forward primer: 5´- tcccggtgacggtgtatgat-3´, reverse primer: 5´- tgccctccttccgcttaaac-3´, probe: 5´- atgaagacgactccccgcccacc-3´

rat Synaptophysin: forward primer: 5´- gcagtgggtctttgccatctt-3´, reverse primer: 5´- tgagggcactctccgtcttg-3´, probe: 5´- cctttgctacgtgtggcagctaca-3´

rat HPRT: forward primer: 5´- cgccagcttcctcctcaga-3´, reverse primer: 5´- ggtcataacctggttcatcact-3´, probe: 5´- ttttcccgcgagccgaccgg-3´
